# Loss of promoter IV-driven BDNF expression impacts oscillatory activity during sleep, sensory information processing and fear regulation

**DOI:** 10.1038/tp.2016.153

**Published:** 2016-08-23

**Authors:** J L Hill, N F Hardy, D V Jimenez, K R Maynard, A S Kardian, C J Pollock, R J Schloesser, K Martinowich

**Affiliations:** 1 Lieber Institute for Brain Development, Johns Hopkins Medical Campus, Baltimore, MD, USA; 2 Sheppard Pratt-Lieber Research Institute, Inc., Baltimore, MD, USA; 3 Department of Psychiatry and Behavioral Sciences, Johns Hopkins University School of Medicine, Baltimore, MD, USA; 4 Department of Neuroscience, Johns Hopkins University School of Medicine, Baltimore, MD, USA

## Abstract

Posttraumatic stress disorder is characterized by hyperarousal, sensory processing impairments, sleep disturbances and altered fear regulation; phenotypes associated with changes in brain oscillatory activity. Molecules associated with activity-dependent plasticity, including brain-derived neurotrophic factor (BDNF), may regulate neural oscillations by controlling synaptic activity. BDNF synthesis includes production of multiple Bdnf transcripts, which contain distinct 5′ noncoding exons. We assessed arousal, sensory processing, fear regulation and sleep in animals where BDNF expression from activity-dependent promoter IV is disrupted (Bdnf-e4 mice). Bdnf-e4 mice display sensory hyper-reactivity and impaired electrophysiological correlates of sensory information processing as measured by event-related potentials (ERP). Utilizing electroencephalogram, we identified a decrease in slow-wave activity during non-rapid eye movement sleep, suggesting impaired sleep homeostasis. Fear extinction is controlled by hippocampal–prefrontal cortical BDNF signaling, and neurophysiological communication patterns between the hippocampus (HPC) and medial prefrontal cortex (mPFC) correlate with behavioral performance during extinction. Impaired fear extinction in Bdnf-e4 mice is accompanied by increased HPC activation and decreased HPC–mPFC theta phase synchrony during early extinction, as well as increased mPFC activation during extinction recall. These results suggest that activity-dependent BDNF signaling is critical for regulating oscillatory activity, which may contribute to altered behavior.

## Introduction

Investigating electrophysiological changes in neural and oscillatory activity in behaving animals allows researchers to study circuit dysfunction in disease models. Neural oscillations are studied in behaving animals by recording electroencephalogram (EEG) or local field potentials (LFPs), and reflect summations of oscillatory activity from surrounding neuronal ensembles.^1^ Coordinated oscillations facilitate communication across brain regions, and their synchronization in various frequency ranges influences neuronal networks that control cognition and behavior.^2^ Distinct patterns of synchronized brain oscillatory activity occur during sensory information processing^3, 4^ and during cortical slow-wave activity (SWA) in non-rapid eye movement (NREM) sleep.^5, 6^ Changes in the magnitude and synchrony of oscillatory activity are linked to multiple psychiatric disorders.^2, 7^ As similar oscillatory activity can be recorded across species, investigating neural oscillatory activity may be a useful translational tool for better understanding the disease etiology. Plasticity molecules contribute to coordination and timing synchronized oscillatory activity by influencing synaptic strength and balancing synaptic inhibition versus excitation.^8, 9^ Hence, disruptions in expression of brain-derived neurotrophic factor (BDNF), which has a key role in regulating synaptic plasticity in the brain, may influence oscillatory activity. However, a link between BDNF signaling, regulation of oscillatory activity and behavioral performance has not been thoroughly established.

The mechanisms by which coordinated brain oscillatory activity contributes to sensory processing and sleep, as well as more complex behaviors including fear learning and extinction are emerging. Synchronized activity facilitates communication between the amygdala (AMY), hippocampus (HPC) and prefrontal cortex (PFC), and regulates fear memory acquisition and extinction. Specifically, synchronous theta activity between HPC, medial PFC (mPFC) and AMY occurs during states of high fear.^10, 11, 12, 13^ Moreover, coherent theta oscillations between mPFC and AMY during sleep are correlated with fear memory consolidation.^14^ Current findings suggest that AMY–HPC–PFC theta synchrony is associated with high fear, whereas decreased synchrony is associated with fear inhibition.^10, 15^ In rodents, inhibiting fear expression during extinction requires communication between ventral HPC (vHPC) and the infralimbic (IL) subdivision of mPFC;^16^ IL activity subsequently modulates AMY function to control fear expression.^17, 18^ BDNF signaling is a key regulator of fear extinction within these circuits.^19, 20, 21, 22, 23, 24^ Hippocampal BDNF infusion decreases fear expression during extinction^21^ and increases IL neuron firing,^22^ suggesting that coordinated BDNF release in the HPC–mPFC circuit is crucial for successful extinction. Furthermore, genetic alterations that result in decreased activity-dependent BDNF release or virally mediated HPC BDNF deletion cause impaired fear extinction in mice.^19, 23^

As many as nine differentially regulated 5′ promoters control spatial and temporal transcription of the *Bdnf* gene.^25^ *Bdnf* promoter IV is strongly induced in response to neuronal activity, and activity-dependent expression of exon-IV-containing transcripts are critical for hippocampal long-term potentiation (LTP) and formation of fear extinction memories.^24, 26, 27^ Although a human genetic mutation that specifically affects production of BDNF from promoter IV has not been identified, evidence suggests a relationship between stress exposure and epigenetic regulation of *BDNF* promoter IV with the development of psychiatric disorders. Specifically, changes in *BDNF* promoter IV methylation levels are implicated in depression.^28, 29^ In addition, exposure to stressful life events has been correlated with increased risk for the development of posttraumatic stress disorder (PTSD) in individuals with a polymorphism in the *BDNF* gene that results in decreased activity-dependent BDNF release.^30, 31, 32^ Although molecules that regulate activity-dependent synaptic plasticity have a role in generating and timing oscillatory activity, studies have not specifically linked activity-dependent BDNF signaling with oscillations that control sensory processing and complex behavior. We assessed how selective disruption of BDNF from activity-dependent promoter IV (Bdnf-e4 mice)^33^ impacts arousal, sensory processing, fear regulation and sleep; phenotypes that are associated with altered brain physiology in PTSD.^2, 34, 35, 36^ Using a combination of behavior testing and *in vivo* electrophysiology, we demonstrate a number of phenotypes relevant to PTSD including sensory hyper-reactivity and impaired neural correlates of sensory information processing as measured with event-related potential (ERP) recordings in Bdnf-e4 mice. EEG recordings in these mice revealed decreased SWA during NREM sleep, suggesting impaired sleep homeostasis. To probe physiological correlates of impaired fear regulation, we conducted LFP recordings in mPFC and HPC during fear recall and extinction. Bdnf-e4 mice show elevated HPC theta power and decreased mPFC–HPC theta phase synchrony concomitant with exaggerated fear during early extinction. The findings suggest that BDNF expression from promoter IV contributes to the physiological mechanisms underlying sensory information processing, sleep homeostasis and fear regulation.

## Materials and methods

### Animals

Bdnf-e4 mice were generated as previously described^33^ (Supplementary Figures S1a–c). Briefly, disruption of BDNF production from promoter IV was achieved by inserting an enhanced green fluorescent protein-STOP cassette such that transcription initiated from promoter IV produces green fluorescent protein in lieu of BDNF (Supplementary Figure S1c). The male mice were maintained on a 12 h light/dark cycle with food and water available *ad libitum*, housed three to five per cage and 10–16 weeks old at the time of experiments. Similar to previous work, groups of *n*=8 or greater were used for behavioral testing.^27, 33, 37^ For electrophysiological measurements, groups of *n*=7 or greater were used, as this group size has been shown to be sufficient to detect group changes.^38, 39^ All the procedures were in accordance with the NIH Guide for the Care and Use of Laboratory Animals and approved by the institutional animal care and use committee.

### Startle/prepulse inhibition

Testing was conducted using the SR-Lab System (San Diego Instruments, San Diego, CA, USA). The animals were first placed in the test cylinder for a 5 min acclimation period. For startle, sounds of five different decibels (dB)—80, 90,100, 110 and 120 dB were played in a pseudo-random order. For prepulse inhibition (PPI), four trial types were conducted-pairing two different decibels (75, 90) with two different interstimulus intervals (50 or 450 ms prepulse). Intertrial order was randomized with 5–20 s between each trial.

### Surgeries

The mice received inhaled isoflurane during all surgeries. For EEG and ERP studies, prefabricated headmounts (catalog #8201, Pinnacle Technologies, St Lawrence, KS, USA) consisting of a ground and reference on opposite hemispheres in the two anterior positions (+3.3 anteroposterior (AP), ±2.0 mediolateral (ML)) and the ground and reference in the posterior positions were implanted. Nuchal leads were inserted for electromyogram recordings. For LFP recordings, custom headmounts containing bipolar twisted leads (35 μm stainless steel wire, California Fine Wire, Grover Beach, CA, USA) were used as depth electrodes and stainless steel screws were used as ground and reference electrodes. Depth electrodes were targeted to IL (+1.94 AP; −0.275 ML; −2.25 mm dorsoventral) and CA1 (−2.92 AP; −3.00 ML; −1.62 mm dorsoventral). Ground and reference screws were placed in the cerebellum (−5.5 AP; 0 ML) and frontal cortex (+1.94 AP; +1.5 ML). The mice received analgesic (1 mg ml^−1^ meloxicam, intraperitoneal) for 3 days and the experiments were conducted 1 week post surgery.

### *In vivo* electrophysiology

The animals were habituated to the tether in a homecage environment for 30 min per day for 2 days. EEG and ERP recordings were conducted in a sound-attenuated chamber with data collected at 1000 Hz. For EEG studies, the mice were recorded for 48 h in a homecage environment with only the final 24 h included in the final analysis. The ERP session was 20 min, with a 5 min acclimation. Auditory stimuli (two 70 dB tones—10 ms in length, 500 ms apart for 100 trials) were generated with Spike2 (Cambridge Electronic Design, Cambridge, UK). For recordings conducted during fear extinction trials, a custom version of FreezeScan (CleverSys, Reston, VA, USA) simultaneously captured and tagged both behavior and electrophysiology in one file, at a rate of 2000 Hz.

### Combined cue/context extinction paradigm

Fear conditioning and extinction were conducted in standard conditioning boxes utilizing FreezeScan (CleverSys) according to the protocol shown in Supplementary Figures S5e–g. Conditioning consisted of two 30 s, 1000 Hz neutral tones, followed by three conditioned stimulus (CS)/unconditioned stimulus pairings (4000 Hz tone, 30 s, 2 s footshock of 0.6 mA at the end of tone). The mice underwent two extinction sessions (20 CS tones, 30 s long with a 5 s intertrial interval, spaced 1 h apart) on day 1 (extinction 1 and 2) and day 2 post conditioning (extinction 3 and 4). On day 3 post conditioning, the animals performed an extinction test (20 CS tones) to examine retention. For extinction 1–4 and extinction test, the percentage of time freezing was averaged across cue presentations.

### Electrode localization

The mice with implanted electrodes were transcardially perfused with 4% paraformaldehyde and brains post-fixed and cryo-protected. Electrode placement was verified by cresyl violet staining on 50 μm coronal sections at × 10 magnification. For representative images (Supplementary Figure S6), brightfield images were acquired at × 2 magnification using an Olympus (Center Valley, PA, USA) BX51TF microscope with DP70 color camera. To reconstruct the coronal section, images were montaged using Neurolucida software (MicroBright Field Bioscience, Williston, VT, USA). Mice with improper placements based on the described coordinates were excluded from further analysis.

### Electrophysiology data analysis

The data collected during ERP recordings and fear extinction trials were processed using EEGLab toolbox (Schwartz Center for Computational Neuroscience) and custom scripts in Matlab (Mathworks, Natick, MA, USA).^40^ For ERP studies, 100 epochs (−0.5 to 1 s relative to tone onset) were analyzed. Grand average data were obtained by averaging single-trial epochs. For the analysis of mean amplitude and latency, data were averaged across the following time windows (sound 1: P1, 5–25 ms; N1, 26–46 ms; P2, 60–80 ms; P3, 100–130 ms; sound 2, the P2 window was adjusted to 50–75 ms, and the P3 was 80–100 ms). For time–frequency analysis, event-related spectral perturbation (ERSP), and intertrial phase coherence (ITC) were calculated using Morlet wavelets in 50 linearly spaced bins between 3 and 50 Hz, with wavelet cycles increasing from 2 to 10. ERSP was measured in dB relative to baseline power (−500 to 0 ms post tone), similar to previously reported.^41^ For experiments conducted during fear extinction sessions, a similar analysis, using log-spacing to better resolve theta activity was applied (−2000 to 2000 ms post-freezing onset). Baseline power was obtained from −700 to −200 s before freezing onset. HPC–mPFC phase cross-channel coherence (CCC) was computed using the cross(*f*) function in EEGLab,^40, 42^ using the same parameters as those reported for spectral analysis. Extracted ERSP and CCC data were averaged across 3–5 Hz and compared between groups. For EEG recordings, wake, NREM and rapid eye movement (REM) sleep states were visually scored from the EEG and electromyogram in 10 s epochs. EEG spectral analyses were performed using custom-programmed tools in Python utilizing the SciPy and Matplotlib libraries and toolboxes. Spectral analysis of SWS, REM and wake periods were performed on concatenated data with the same classification score for a single animal for a given 24 h period using the power spectral density function of matplotlib, which uses the Welch method. A moving window of 2048 ms was used, with an equal number of fast fourier transforms and 50% overlap. The data were then averaged across each group.

### Statistics

Statistics were computed with GraphPad Prism Software (La Jolla, CA, USA). The data with single averaged values for genotype were compared using an unpaired Student's *t*-test, including ERP components, total time spent conducting specific behaviors, as well as ERSP and CCC data. The behavioral data comparing genotypes across different trial types or time points used two-way repeated-measures analysis of variance, including ERP time–frequency results, sleep/wake state duration per hour, sleep/wake EEG power and time freezing during extinction CS presentation. *Post hoc* Bonferroni's multiple comparisons were carried out where applicable. The reported data were normally distributed and are presented as mean±s.e.m.

## Results

### Bdnf-e4 mouse model

The mice that were previously generated to selectively disrupt BDNF expression from activity-dependent promoter IV (BDNF-KIV mice) showed deficits in GABAergic signaling, which were linked to fear extinction and reversal learning deficits.^27, 43^ As follow-up studies identified downregulation of alternate *Bdnf* transcripts in BDNF-KIV mice,^37^ we utilized an updated line (Bdnf-e4) that was designed to correct downregulation of alternative transcripts to confirm and extend findings on the role of promoter IV-driven BDNF expression (Supplementary Figures S1a–c).^33^ In the Bdnf-e4 mice, the engineered enhanced green fluorescent protein-STOP cassette results in a complete blockade of BDNF production from promoter IV, with green fluorescent protein produced in lieu of BDNF (Supplementary Figure S1c). Quantitative PCR confirmed the loss of *Bdnf* exon-IV-containing transcripts in Bdnf-e4 PFC and HPC (Supplementary Figure S1d). We identified minor, although significant decreases in *Bdnf* exon I-containing transcripts in HPC (Supplementary Figure S1d, right); however, as discussed in the previous characterization of the Bdnf-e4 line,^33^ this likely reflects downstream biological regulation rather than a confounded targeting strategy. Similar to BDNF-KIV, we found significant downregulation of interneuron markers in Bdnf-e4 PFC and HPC (Supplementary Figure S1e). Differing from BDNF-KIV mice,^37, 44^ analyses of baseline locomotion, anxiety and depressive-like behavior, as well as homecage behavior revealed no differences between wild-type (WT) and Bdnf-e4 mice (Supplementary Figures S2a–h). Differences in behavior between the two lines may arise from the decreases in transcription from alternative *Bdnf* promoters observed in BDNF-KIV that are not observed in Bdnf-e4 mice.^37^

### Deficits in sensory information processing in Bdnf-e4 mice

**Figure 1:**
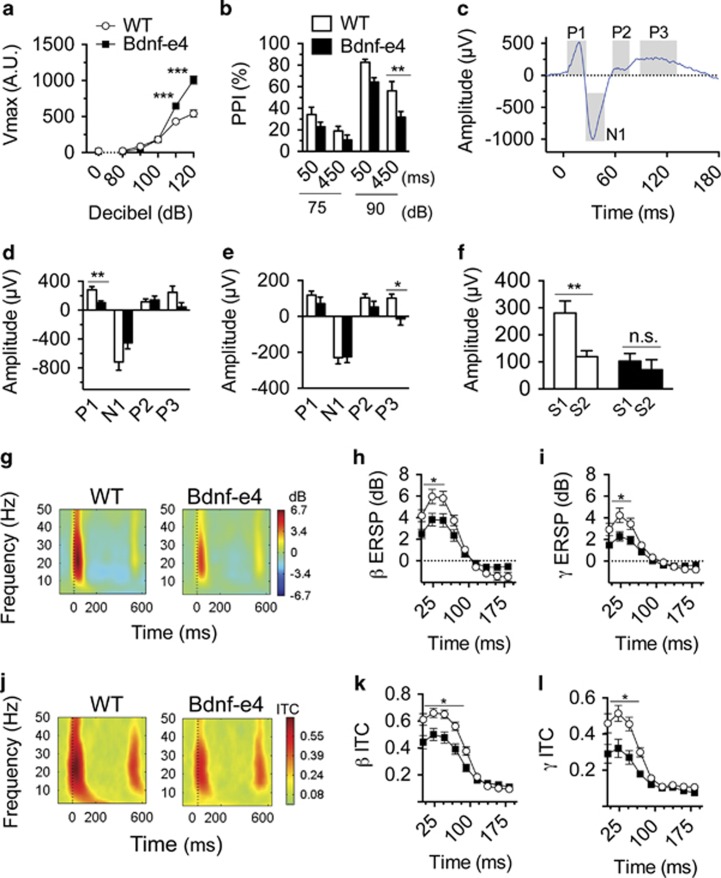
Sensory information processing deficits in Bdnf-e4 mice. (**a**) Differences in startle reactivity were revealed by two-way analysis of variance (ANOVA) as a significant effect of genotype (F_1,17_=31.33; *P*<0.0001) and an interaction between genotype and decibel (dB) intensity (F_5,85_=24.78; *P*<0.0001). *Post hoc* analysis revealed significantly increased response to 110 (*P*<0.0001) and 120 dB (*P*<0.0001) tones in Bdnf-e4 mice (*n*=10) compared with wild-type (WT; *n*=9). (**b**) The differences in prepulse inhibition (PPI) were observed in Bdnf-e4 mice (*n*=10) compared with WT (*n*=10) by a two-way ANOVA as a significant effect of genotype (F_1,18_=6.673; *P*=0.0187), and *post hoc* analysis showed that Bdnf-e4 mice displayed significantly less PPI in the 90 dB/450 ms trial (*P*<0.01). (**c**) Example event-related potential (ERP) grand average with analyzed components (P1, N1, P2 and P3). Time 0=Sound 1 (S1) onset, shaded areas represent the time periods averaged for mean amplitude analysis of each component. (**d**) In response to S1, the P1 mean amplitude is decreased in Bdnf-e4 mice compared with WT (*n*=8, each genotype), with trends towards decreased N1 and P3 (P1: *P*=0.0048; N1: *P*=0.0794; P3: *P*=0.0790, Student's *t*-test). (**e**) Following Sound 2 (S2), the P3 mean amplitude was significantly decreased in Bdnf-e4 mice (P3: *P*=0.0197, Student's *t*-test). (**f**) The differences in S1 and S2 amplitudes of the P1 component were revealed by two-way ANOVA as a significant effect of genotype (F_1,14_=7.532, *P*=0.0158) and an interaction between genotype and stimulus trial (F_1,14_=6.7444, *P*=0.0211). *Post hoc* analysis showed intact P1 gating in WT animals as a significant difference between P1 amplitudes for S1 and S2 in WT (*P*<0.001), but not in Bdnf-e4 mice. (**g**) Heat maps of event-related spectral perturbation (ERSP) in WT (left) and Bdnf-e4 (right) depict ERP-related changes in electroencephalogram (EEG) power with time=0 representing tone onset. (**h** and **i**) Two-way ANOVA revealed significantly different averaged ERSP for 0–200 ms post tone between WT and Bdnf-e4 mice in (**h**) beta (β) frequency (12–30 Hz; genotype × time interaction, F_52,728_=5.709; *P*<0.0001; *post hoc t*-tests, 16–46 ms, *P*<0.05) and (**i**) gamma (γ) frequency (30–50 Hz; genotype × time interaction, F_52,728_=4.126; *P*<0.0001; *post hoc t*-tests, 8–42 ms, *P*<0.05). (**j**) Heat maps of intertrial coherence (ITC) in WT (left) and Bdnf-e4 (right) depict ERP-related changes in phase coherence. (**k** and **l**) Two-way ANOVA revealed significantly different averaged ITC between WT and Bdnf-e4 mice in (**k**) beta (β) frequency (genotype, F_1,14_=7.198, *P*=0.0178 and genotype × time interaction, F_52,728_=10.02, *P*<0.0001; *post hoc t*-tests, 1–88 ms, *P*<0.05) and (**l**) gamma (γ) frequency (genotype, F_1,14_=11.04; *P*=0.0050) and genotype × time interaction (F_52,728_=6.504; *P*<0.0001; *post hoc t*-tests, 1–65 ms, *P*<0.05). The data are represented as mean±s.e.m. (*P<0.05, **P<0.001, ***P<0.0001, NS, not significant). Bdnf, brain-derived neurotrophic factor.

Heightened auditory stimulus-evoked startle and deficits in PPI reflect disruptions of sensory processing and sensorimotor gating that are observed in patients with PTSD.^34, 45^ Compared with WT, the Bdnf-e4 mice display an exaggerated acoustic startle response (Figure 1a), and decreased PPI (Figure 1b).

ERPs are stereotyped patterns of voltage fluctuation measured in response to sensory stimuli, comprising individual components that reflect temporal aspects of the physiological response. An inhibited physiological response to a second, similar stimulus is referred to as gating.^3, 46^ To assess the neurophysiological basis of sensory processing deficits in Bdnf-e4 mice, we conducted two-tone auditory ERP recordings that allowed us to assess individual ERP components (P1, N1, P2 and P3) and gating (Figure 1c, Supplementary Figure S3a). Mean P1 amplitude in response to sound 1 (S1) was significantly decreased in Bdnf-e4 mice compared with WT, with trends towards decreased N1 and P3 (Figure 1d, Supplementary Figure S3b). There was no genotype-related difference in S1 or sound 2 (S2) peak amplitude latency (data not shown). For S2, there was no difference in the early components, but a difference in the P3 mean amplitude (Figure 1e, Supplementary Figure S3c) existed. WT animals strongly gated the P1 response; however, there was no decrease in P1 amplitude from S1 to S2 in Bdnf-e4 mice (Figure 1f).

As event-related power and phase locking are hypothesized to reflect strength and connectivity in cortical circuits, we used time–frequency analysis of the ERP to gain insight into the underlying brain activity and circuitry.^3^ We evaluated ERSP power changes relative to tone onset in Bdnf-e4 and WT mice (Figures 1g–i). We also evaluated phase-locking changes by examining group differences in ITC (Figures 1j–l). For both ERSP and ITC measures, the frequency with the largest magnitude of change in S1 response in WT animals occurred in the beta range (12–30 Hz). There were no genotype differences in time–frequency results specific to S2; for this reason only data from the first 200 ms following S1 are shown. Following S1, we found significantly decreased ERSP and ITC beta frequency response in Bdnf-e4 mice compared with WT (Figures 1h and k). The ERSP and ITC were also both significantly decreased in the gamma frequency range (30–50 Hz, Figures 1i and l). Although the overall magnitude of response in lower frequency ranges was decreased in comparison with beta and gamma, there were additional decreases in the Bdnf-e4 ERP response to S1 in both the theta and alpha frequency ranges (Supplementary Figures S3d–g).

### Altered SWA in Bdnf-e4 mice

**Figure 2:**
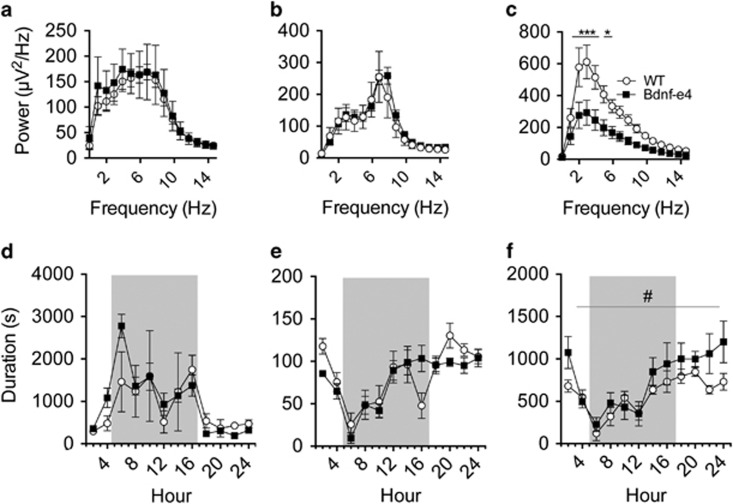
Sleep architecture and physiology in Bdnf-e4 mice. (**a**–**c**) Averaged power spectra presented for Bdnf-e4 (*n*=8) and wild-type (WT) mice (*n*=7) during identified wake, rapid eye movement (REM) and non-REM (NREM) sleep states. There was no difference in (**a**) average power during wake or (**b**) average power during REM sleep. (**c**) Bdnf-e4 mice exhibit significantly less slow-wave activity (SWA) power during NREM sleep compared with WT. Two-way analysis of variance (ANOVA) revealed a difference between genotypes (F_1,819_=129.8, *P*<0.0001) as well as a genotype × frequency interaction (F_62,819_=3.703, *P*<0.0001). *Post hoc* analysis revealed significantly decreased power at frequencies 1.5–5.9 Hz (*post hoc t*-tests, 1.5–4.4 Hz, *P*<0.0001, 4.9–5.9, *P*<0.05). (**d**–**f**) Average time spent in each sleep state, hour by hour across the circadian cycle for WT (*n*=7) and Bdnf-e4 mice (*n*=8). Shaded portions indicate lights off. There are no differences (**d**) in the time spent awake per hour or (**e**) in the time spent in REM sleep per hour. (**f**) Bdnf-e4 mice spent significantly more time per hour in NREM, as demonstrated by a two-way ANOVA genotype difference (F_1,297_=9.744, *P*=0.002). Data are represented as means±s.e.m. (*P<0.05, ***P<0.0001; genotype difference, ^#^*P*<0.01). Bdnf, brain-derived neurotrophic factor.

Sleep dysfunction reflects underlying circuit malfunction, and is also linked to abnormal synaptic plasticity.^47, 48, 49^ Prolonged wakefulness increases synapse strength,^50^ and promotes cortical SWA,^38^ the EEG power between 0.5–4.5 Hz. Importantly, the magnitude of SWA has been causally linked to cortical BDNF expression.^51, 52^ People with disordered sleep are at increased risk for developing PTSD,^53^ and individuals with PTSD exhibit decreased SWA.^54, 55^ To investigate how loss of *Bdnf* exon-IV-containing transcripts impacts sleep physiology, we performed EEG recordings in a homecage environment with Bdnf-e4 and WT mice (experimental details in Supplementary Methods). We analyzed the power spectra during identified time in wake, REM or NREM sleep states (Figures 2a–c, Supplementary Figure S4). We found no significant between-group differences in wake-associated power (Figure 2a) or REM power (Figure 2b). However, Bdnf-e4 mice had significantly decreased power compared with WT in the delta/low theta frequency range during NREM sleep (Figure 2c). Although there were no changes in time spent awake or in REM sleep (Figures 2d and e), Bdnf-e4 mice exhibited an increase in time spent in NREM per hour (Figure 2f), which could reflect an attempt to compensate for decreased SWA activity.

### Impaired fear regulation in Bdnf-e4 mice

Previous work revealed deficits in contextual, but not cued extinction in BDNF-KIV mice.^27^ To confirm this finding in Bdnf-e4 mice, we assessed extinction of both contextual and cued fear. In all the experiments, the mice were conditioned using three unconditioned stimulus (footshock)/CS (tone) pairings. Confirming normal fear acquisition, time spent freezing following conditioning was equivalent between genotypes in both experiments (Supplementary Figures S5a and c). During cued fear extinction, there were no differences in time spent freezing between WT and Bdnf-e4 mice during CS presentation (Supplementary Figure S5b). However, during context extinction, there was a significant increase in time spent freezing during CS presentation in Bdnf-e4 mice (Supplementary Figure S5d).

**Figure 3:**
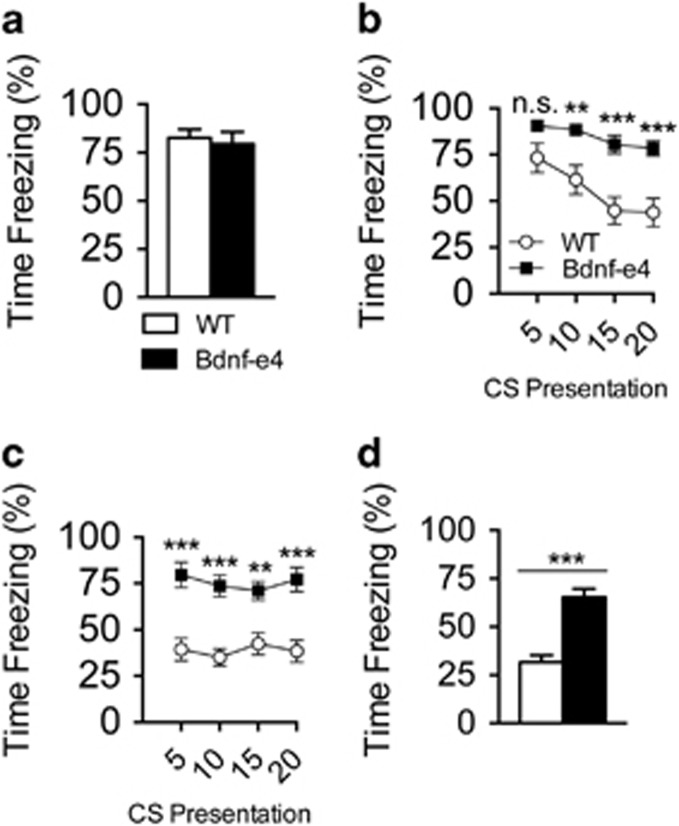
Bdnf-e4 mice display behavioral impairment in fear extinction. (**a**) Time spent freezing during conditioning. There was no difference between wild-type (WT; *n*=10) and Bdnf-e4 mice (*n*=11) in the time spent freezing in the last 30 s of conditioning. (**b**) Within-session freezing behavior during extinction 1. Each point represents the average percentage of time spent freezing during five conditioned stimulus (CS) presentations. Two-way analysis of variance (ANOVA) data demonstrate a significant effect of genotype on freezing behavior (F_1,19_=18.79, *P*=0.0004). *Post hoc* analysis confirmed no significant difference in average time spent freezing between WT and Bdnf-e4 mice during CS presentation 1–5; however, in subsequent CS presentations, Bdnf-e4 mice spent significantly more time freezing (CS=6–10, *P*<0.001; CS=11–15, *P*<0.0001; CS=16–20, *P*<0.0001). (**c**) Within-session freezing behavior during extinction 3. Two-way ANOVA data demonstrates a significant effect of genotype on freezing behavior (genotype effect; F_1,19_=24.54, *P*<0.0001). *Post hoc* analysis demonstrates that increased time spent freezing by Bdnf-e4 mice was significant across the trial (CS=1–5, 6–10 and 16–20, *P*<0.0001; CS=11–15, *P*<0.001). (**d**) Extinction test on day 3 freezing behavior averaged, demonstrating Bdnf-e4 mice spend significantly more time freezing during extinction recall (Student's *t*-test, *P*<0.0001). Data are represented as means±s.e.m. (**P<0.001, ***P<0.0001, NS, not significant). Bdnf, brain-derived neurotrophic factor.

We next assessed fear extinction in a combined cue/context paradigm. In this paradigm, extinction was divided into four sessions following conditioning, with two sessions conducted 24 h post conditioning and additional two sessions 48 h post conditioning. During each extinction session, mice were returned to the conditioning chamber and re-exposed to the CS. Seventy-two hours post conditioning, an extinction test session was conducted to probe extinction recall (Supplementary Figures S5e–g). Although there was no difference in time spent freezing following conditioning (Figure 3a), Bdnf-e4 mice exhibited impaired fear extinction during all extinction sessions, as well as during the extinction test (Figures 3b–d). To gain insight into the dynamics of within-session freezing, we analyzed average time spent freezing across groups of five CS presentations for extinction 1 and extinction 3 (Figure 3b and c). Importantly, although Bdnf-e4 mice froze significantly more overall during extinction 1, *post hoc* analysis confirmed no genotype difference in time spent freezing during CS 1–5 at the beginning of extinction. This suggests that there was not a difference in the initial fear recall following acquisition. During extinction 3, time spent freezing remained significantly higher in Bdnf-e4 mice (Figure 3c). Bdnf-e4 mice continued to spend more time freezing during the extinction test (Figure 3d).

### Bdnf-e4 mice show increased HPC and mPFC theta frequency activity during fear recall and extinction

**Figure 4:**
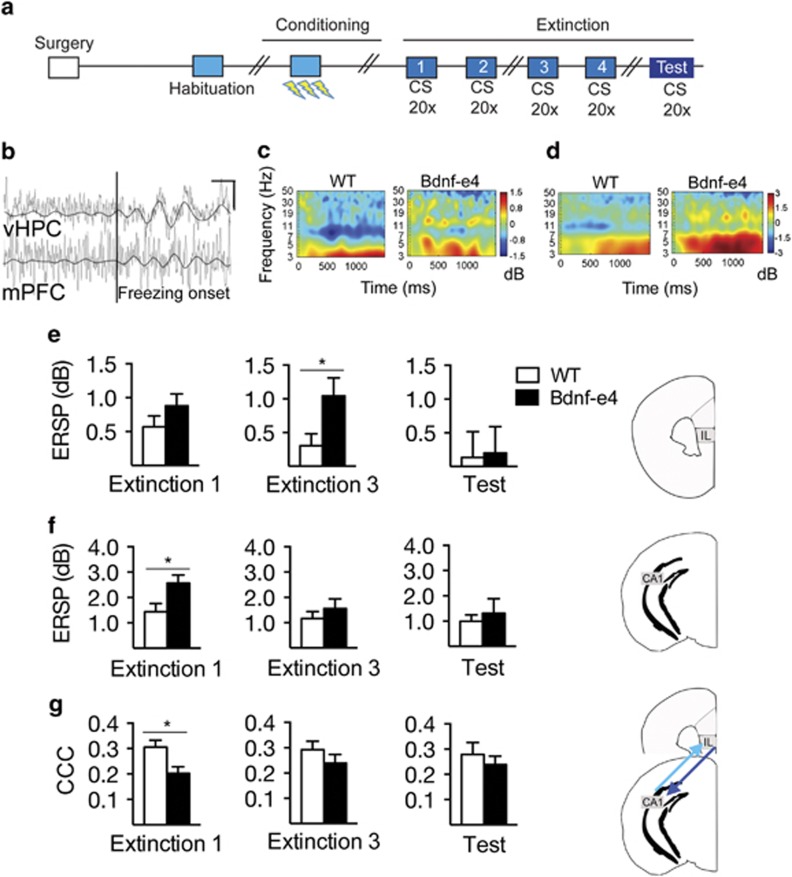
Bdnf-e4 mice exhibit changes in low theta (3–5 Hz) frequency power and synchrony during extinction. (**a**) Schematic of experimental design. (**b**) Representative example of raw local field potential (LFP) trace from the CA1 region of the hippocampus (HPC) and the infralimbic (IL) region of medial prefrontal cortex (mPFC) recorded during extinction 1, overlaid with a 3–5 Hz filter to emphasize increase in low theta at freezing onset (indicated by perpendicular black line). Scale bars, vertical=90 μV, horizontal=200 ms (upper right hand quadrant). Heat map depicting event-related spectral perturbation (ERSP) data for both WT and Bdnf-e4, measured in decibels (dB), averaged by freezing onset during extinction 1 for (**c**) mPFC and (**d**) HPC. (**e**–**f**) ERSP group changes in low theta power relative to freezing onset, displayed from all experimental sessions for mPFC and HPC. (**e**) mPFC ERSP response for extinction 1, 3 and extinction test, averaged from 3 to 5 Hz for 0–1000 ms following freezing onset. During extinction 3, Bdnf-e4 mice (*n*=11) have significantly higher mPFC low theta ERSP than WT (*n*=10; Student's *t*-test, *P*=0.0358). (**f**) HPC ERSP response for extinction 1, 3 and extinction test, averaged from 3 to 5 Hz. During extinction 1, Bdnf-e4 mice (*n*=10) have significantly higher HPC low theta ERSP than WT (*n*=11; Student's *t*-test, *P*=0.0238). (**g**) Cross-channel coherence (CCC) measurement to examine differences in low theta phase synchrony between HPC and mPFC during 0–1000 ms post freezing across extinction 1, 3 and extinction test. In addition to the significant increase in HPC low theta power in extinction 1, there is also a significant difference in CCC between WT (*n*=9) and Bdnf-e4 mice (*n*=10). Bdnf-e4 mice have significantly lower theta CCC compared with WT (Student's *t*-test, *P*=0.0140). Data are represented as means±s.e.m. (*P<0.05).

BDNF release in the HPC to mPFC projection is implicated in extinction learning,^21^ and HPC BDNF infusion increases firing in IL cortex,^22^ contributing to successful extinction. To determine whether Bdnf-e4 mice display altered patterns of spectral power during fear extinction, we targeted electrodes to the IL region of mPFC and the CA1 region of HPC (Figure 4a, Supplementary Figure S6). After surgical recovery and fear conditioning, we acquired LFP recordings from animals performing extinction sessions 1–4 and the extinction test (Figure 4b, Supplementary Figure S7). There was no difference between genotypes in the number of freezing events used for spectral analysis within a session (Supplementary Figure S7d). Increased time spent freezing in Bdnf-e4 mice is accounted for by an increased duration of freezing during each freezing episode compared with WT (Supplementary Figure S7e).

We examined changes in spectral power during freezing relative to a pre-freezing baseline, and generated heat maps for freezing-associated changes in mPFC and HPC LFP power for WT and Bdnf-e4 mice (Figures 4c and d). As evidenced by the heat maps, WT mice showed a relative decrease in power in the ~7–12 Hz theta range, and a significant increase in the 3–5 Hz range during freezing behavior. Similar power changes during freezing have previously been described.^56^ For each extinction session, low theta (3–5 Hz) ERSP data was averaged and compared between WT and Bdnf-e4 mice (Figures 4e and f). During habituation, there were no genotype differences in HPC and mPFC ERSP data nor in HPC–PFC coherence (Supplementary Figures S8a–c). Both genotypes showed increased power in the low theta frequency range in mPFC and HPC during extinction 1, but HPC low theta power during freezing behavior was significantly higher in Bdnf-e4 mice relative to WT (Figure 4f, left). The average HPC low theta power for extinction 3 was similar between WT and Bdnf-e4 (Figure 4f, center); however, there is a genotype × time interaction when the data is examined across an extended period of the post-freezing time in extinction 3 (Supplementary Figure S8e). In addition to this increase in HPC low theta power, Bdnf-e4 mice also showed increased low theta power in the mPFC compared with WT during extinction 3 (Figure 4e, Supplementary Figure S8d). By the extinction test on the third day post conditioning, no between-group ERSP power differences remained (Figures 4e and f, right).

### Bdnf-e4 mice show decreased HPC–mPFC phase coherence during extinction

In addition to changes in the magnitude of low theta power, phase-synchronization of theta activity is an important physiological correlate of behavior in fear memory recall and extinction learning tasks.^10, 11, 15^ Extinction-related disturbances in theta synchrony are linked to impaired extinction behavior.^11, 12, 13^ Specifically, theta activity is synchronized during early extinction when fear expression is high, and decreases across the course of extinction as fear expression attenuates.^10, 15^ Thus, we hypothesized that HPC–mPFC theta synchrony during extinction learning would be impaired in Bdnf-e4 mice. We examined HPC–mPFC synchrony by measuring CCC during freezing epochs, from 0 to 1000 ms following freezing onset. These analyses were conducted using the LFP data acquired in the above spectral analyses. During extinction 1, there was significantly less theta synchrony between HPC and mPFC in Bdnf-e4 mice (Figure 4g, left). Although the difference in average CCC for extinction 3 did not reach significance (Figure 4g, middle), there is a genotype × time interaction when the data are examined across the post-freezing time course during extinction 3 (Supplementary Figure S7f). This suggests that although the magnitude of the group difference decreased by extinction 3, Bdnf-e4 mice retained a HPC–mPFC phase synchrony impairment compared with WT during freezing in this session. By the extinction test, there were no significant group differences in theta synchrony, as measured by CCC (Figure 4g, right).

## Discussion

Brain oscillations provide a mechanism for organizing communication between neuronal ensembles that are functionally co-activated during behavior. Coherent oscillatory activity is required for sensory perception, sleep and fear regulation,^2^ behaviors that are impaired in PTSD,^2, 34, 35, 36^ and linked to BDNF signaling.^8, 9^ Because BDNF signaling has a critical role in synaptic plasticity and regulation of excitatory/inhibitory balance, it is a likely candidate for regulating brain oscillatory activity. However, the hypothesis that BDNF's role in these behaviors is linked to neural synchrony has not been tested. Our ERP, EEG and LFP recordings conducted during extinction in Bdnf-e4 mice suggest that a genetic mutation that selectively targets activity-dependent BDNF signaling contributes to generation of synchronous oscillatory brain activity that is important for regulating sensory processing, sleep and fear behavior.

Behavioral and electrophysiological correlates of sensory perception and information processing are impaired in patients with PTSD.^35, 36^ Individuals with PTSD show alterations in the amplitude, latency and gating of specific ERP components.^34, 35, 45, 57^ Although these deficits are not unique to PTSD, increased arousal is a core symptom of PTSD.^58, 59^ In addition, ERP studies conducted in individuals with PTSD found correlations between altered sensorimotor gating and symptom severity.^34^ In Bdnf-e4 mice, we observed altered sensory processing at the behavioral level as measured by heightened startle and decreased PPI. In addition, at the electrophysiological level, we found impairments in early sensory processing in the Bdnf-e4 mice, as demonstrated by changes in event-related power and intertrial coherence in the beta and gamma range. Sensory processing is coordinated by synchronous activity in the beta frequency range;^60^ hence the observed decrease in beta and gamma activity likely reflect impairments in early processing, consistent with decreased P1/N1. These findings are similar to those obtained from startle reflex testing and ERP recordings in individuals with PTSD.^34, 35, 45, 57^ The decreased P1 gating in Bdnf-e4 mice may be relevant for the observed impairments in fear extinction, as plastic changes in the P1 response occur following fear conditioning and extinction in humans. Post conditioning, P1 gating is reduced during CS replay, and CS-related P1 gating is restored following extinction.^61^ Thus, gating decreases when the presented stimulus is predicting a threat, and returns to normal once an individual no longer associates the CS with an aversive outcome. Hence, impaired gating in Bdnf-e4 mice may decrease their ability to modulate a salience-based response to threat.

Our EEG data demonstrate that loss of promoter-IV-derived BDNF causes decreased SWA during NREM sleep. BDNF is involved in sleep-related synaptic potentiation, and it is argued that wake-related plasticity drives the increase in SWA that occurs following learning.^8, 52^ Indeed, BDNF infusion increases SWA magnitude.^51^ SWA generation relies on cortical neurons being simultaneously depolarized or hyperpolarized, and this synchrony is directly related to synaptic connectivity and efficacy.^62, 63^ At the behavioral level, SWA magnitude is positively correlated with cognitive performance.^64, 65, 66^ A genetic link between activity-dependent BDNF signaling and SWA is established in humans. Individuals with a single-nucleotide polymorphism in the *BDNF* gene that leads to a valine being replaced by a methionine (Val66Met) have decreased activity-dependent BDNF secretion.^32^ Met carriers have decreased SWA during NREM^47, 67^ as well as impaired long-term memory performance following sleep.^47^ Individuals with PTSD exhibit decreased SWA during NREM sleep,^54, 55^ and Met carriers with a PTSD diagnosis experience less symptom improvement following exposure therapy.^20^ Met carriers also demonstrate increased HPC activation during memory testing.^32^ Other studies have found an increase in HPC activity and reduced long-term memory recall following sleep in individuals with decreased SWA and HPC–PFC communication.^66^ Thus, our work contributes to the notion that activity-dependent BDNF signaling contributes to SWA, which may influence extinction learning. Future studies examining EEG activity in the sleep periods following conditioning and extinction in Bdnf-e4 mice may elucidate links between activity-dependent BDNF signaling, sleep and fear regulation.

Given the importance of BDNF in the HPC–IL circuit during fear extinction,^21, 22^ we conducted electrophysiological recordings to examine neural activity within and between these regions during extinction training and recall in Bdnf-e4 mice. We observed an exaggerated increase in low theta frequency power in the HPC of Bdnf-e4 mice during freezing, consistent with HPC overactivation in individuals with the Val66Met polymorphism.^47^ This finding may be due to aberrant HPC–PFC communication, as others have hypothesized that similar findings are a result of decreased consolidation of HPC-based memories during NREM,^66^ or improper HPC engagement during PFC-mediated tasks.^32^ We also observed higher IL low theta activity in Bdnf-e4 mice during extinction 3. A paradoxical increase in IL neuronal firing in mice with impaired extinction has previously been observed.^68^ Specifically, it was hypothesized that IL overactivity may be a compensatory attempt to counteract higher activity in fear-promoting regions, such as prelimbic cortex. It is also possible that low theta activity is tightly linked to the prolonged fear expression in Bdnf-e4 mice, as others have shown that the theta frequency displaying the largest power magnitude in the PFC^56^ and the highest correlation between HPC-AMY^69^ shifts from 8 to 12 Hz during non-freezing behaviors to 4–5 Hz during freezing behavior. In extinction 3, we saw heightened low theta activity in Bdnf-e4 mice in both HPC and mPFC. Thus, promoter IV-derived BDNF impacts the physiological processes that occur during early extinction learning, as well as during subsequent extinction recall. Future work elucidating the genetic and molecular mechanisms that control extinction-related oscillatory activity will be important for advancing our understanding of PTSD, as EEG changes in oscillatory power have been associated with extinction learning in humans.^70^

In addition to changes in theta power, we observed decreased HPC–mPFC theta phase synchrony during extinction 1 and extinction 3 in Bdnf-e4 mice compared with WT. Typically, HPC–PFC–AMY theta synchrony is high during early extinction when fear expression is high and decreases across the course of extinction learning.^10^ Theta synchrony onset follows a distinct time course, which may be due to a requirement for synaptic plasticity. Narayanan *et al.*^71^ demonstrated that between the AMY and HPC, increased theta phase coherence did not emerge until 24 h post conditioning. It was suggested that theta activity is a transient signal of memory reactivation that occurs during long-term memory formation, concurrent with conditioning-induced changes in gene expression. Others demonstrated links between specific genetic manipulations or congenic strains and altered synchronous theta activity.^12, 13, 68, 72^ These findings suggest a functional link between plasticity, theta activity and fear memory expression. Thus, decreased HPC–mPFC synchrony in Bdnf-e4 mice may reflect deficits in learning-induced plasticity that require expression of promoter-IV-derived BDNF. Fear conditioning facilitates CA1 LTP,^73^ and BDNF-KIV mice, a model of decreased activity-dependent BDNF expression, exhibit decreased CA1 late LTP.^27^ A link between decreased LTP and decreased theta phase synchrony is also found in a model of stress-induced model of depression. Specifically, induction of LTP in the HPC–PFC pathway was reduced relative to nonstressed mice, and there was less enhancement in post-LTP HPC–PFC theta phase synchrony.^74^ As carriers of the BDNF Val66Met mutation are at increased risk for PTSD development when exposed to multiple stressful life events,^30, 31^ it would be interesting to explore the relationship of carrier status to theta activity and memory recall in individuals with PTSD.

Our LFP data was acquired in the intermediate/ventral region of the hippocampus (Supplementary Figure S6). In the current literature, the vHPC is often identified as the ventral, posterior one-third of the hippocampus.^75, 76^ vHPC projects directly to both IL and AMY, and carries the majority of outbound communication from the HPC to these regions.^16, 77^ vHPC is important in regulating fear expression and extinction learning.^16^ For instance, mice in which vHPC is pharmacologically inactivated before extinction display reduced conditioned freezing, and poor extinction recall.^78^ The region from which we recorded is in the posterior portion of HPC, but is more dorsal compared with most regions described as vHPC.^11, 22^ The cells in this intermediate region comprises a mixture of cells with some containing projection patterns similar to dorsal HPC (dHPC) and others similar to vHPC.^79, 80^ Similar to vHPC, the intermediate region contains some direct projections to mPFC.^77^ Furthermore, BDNF expression in both dHPC^21, 23^ and vHPC regions^22^ similarly influences fear expression during extinction. Mice with dHPC-specific viral deletion of BDNF exhibit decreased extinction learning compared with controls.^23^ Infusion of BDNF into either dHPC^21^ or vHPC^22^ before extinction attenuates fear expression. Finally, electrophysiological studies suggest that both dHPC and vHPC exhibit changes in theta activity during fear extinction. Specifically dHPC–mPFC theta coherence increases during early extinction learning,^10^ and both dHPC and vHPC theta power increase in response to CS re-exposure.^11^

In conclusion, our results demonstrate that mice with disruption of BDNF expression from promoter IV exhibit electrophysiological changes that correlate with altered behavior. These data are consistent with behavioral observations in PTSD, including heightened startle response, impairments in sensory gating and sleep disturbances. In addition, electrophysiological data acquired during impaired fear extinction suggest that disruption of BDNF signaling leads to changes in HPC and mPFC theta activity. In summary, our findings suggest that activity-dependent BDNF expression via promoter IV is critical in coordinating synchronous oscillatory activity during sensory perception, sleep and memory regulation.
