# Supplementary material for: Loss of promoter IV-driven BDNF expression impacts oscillatory activity during sleep, sensory information processing and fear regulation
Source: Transl Psychiatry. 2016 Aug 23;6(8):e873–. doi: 10.1038/tp.2016.153 (PMC5022093; doi:10.1038/tp.2016.153)
Supplement: Supplementary Information [file tp2016153x1.pdf]

Supporting Information for J. Hill et al. **“Loss of promoter IV-driven BDNF expression impacts oscillatory activity during sleep, sensory information processing and fear regulation”**

Eight supporting figures (Figs. S1 to S8) with accompanying legends and supplementary methods are provided in the Supporting Information.

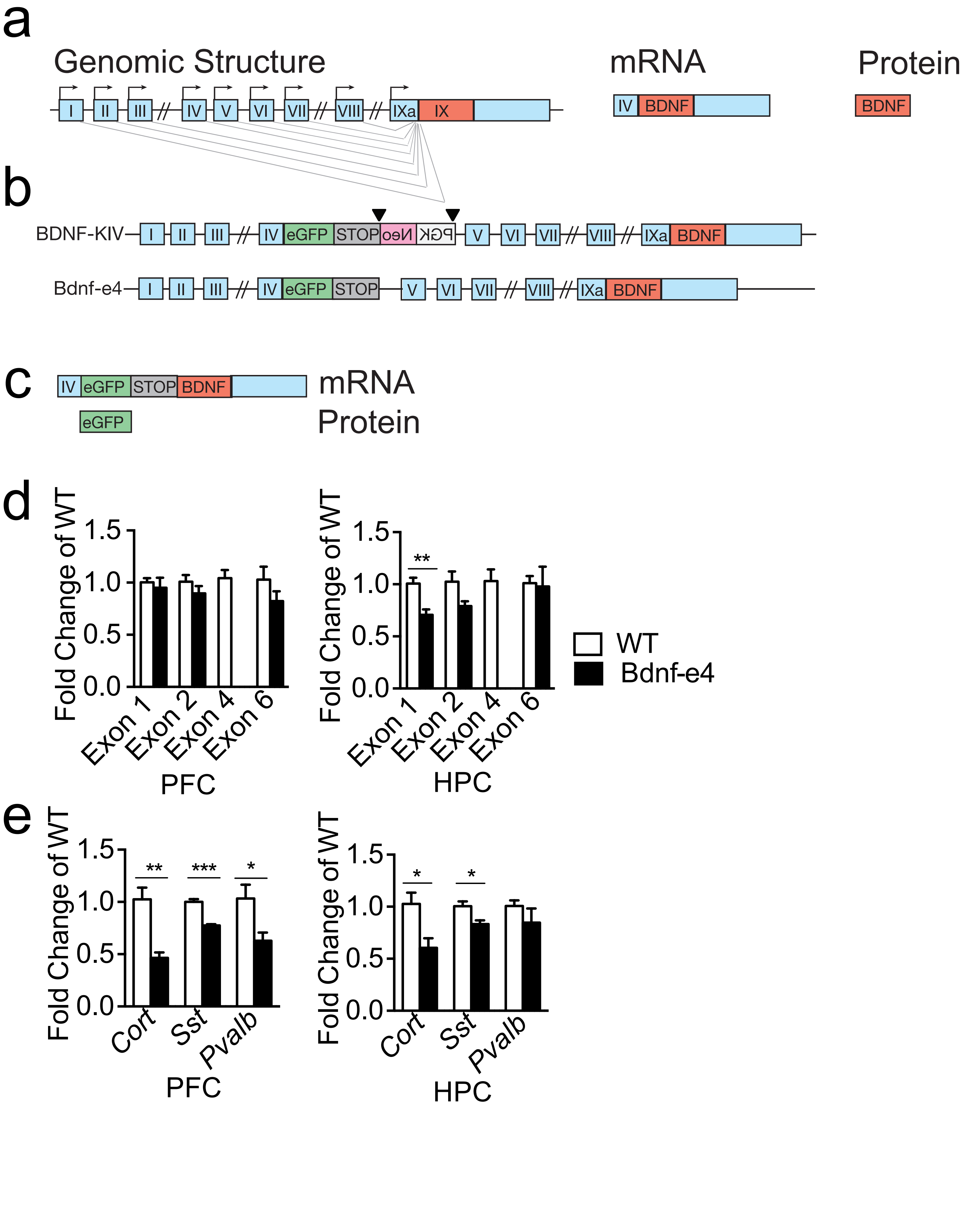
a
Genomic Structure
I
II
III
IV
V
VI
VII
VIII
IXa
IX
mRNA
IV
BDNF
Protein
BDNF
b
BDNF-KIV
I
II
III
IV
eGFP
STOP
Neo
PGK
V
VI
VII
VIII
IXa
BDNF
Bdnf-e4
I
II
III
IV
eGFP
STOP
V
VI
VII
VIII
IXa
BDNF
c
IV
eGFP
STOP
BDNF
mRNA
eGFP
Protein
d
Fold Change of WT
1.5
1.0
0.5
0.0
**
Exon 1
Exon 2
Exon 4
Exon 6
PFC
HPC
WT
Bdnf-e4
e
Fold Change of WT
1.5
1.0
0.5
0.0
**
***
*
Cort
Sst
Pvalb
PFC
*
*
HPC

Figure S1

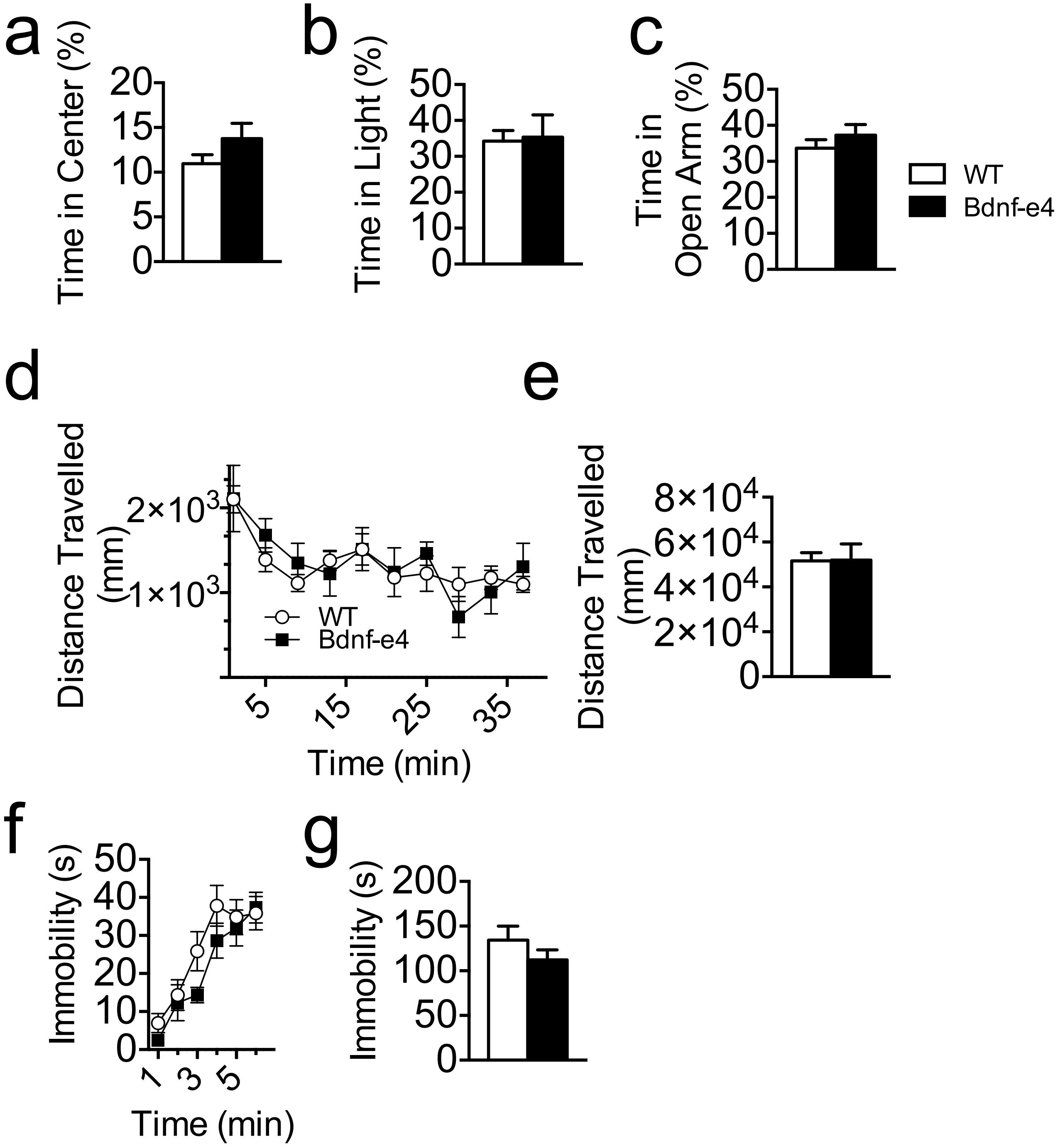

h

Activity	Experimental Group<br>Total Duration Conducting Activity (s)		p-value
	WT	*Bdnf-e4*	
Eat	7065.50	6809.81	0.85
Drink	855.18	812.17	0.74
Rear Up	5072.04	4231.02	0.15
Walk	4580.11	4591.91	0.98
Groom	28191.43	27795.66	0.83

Figure S2

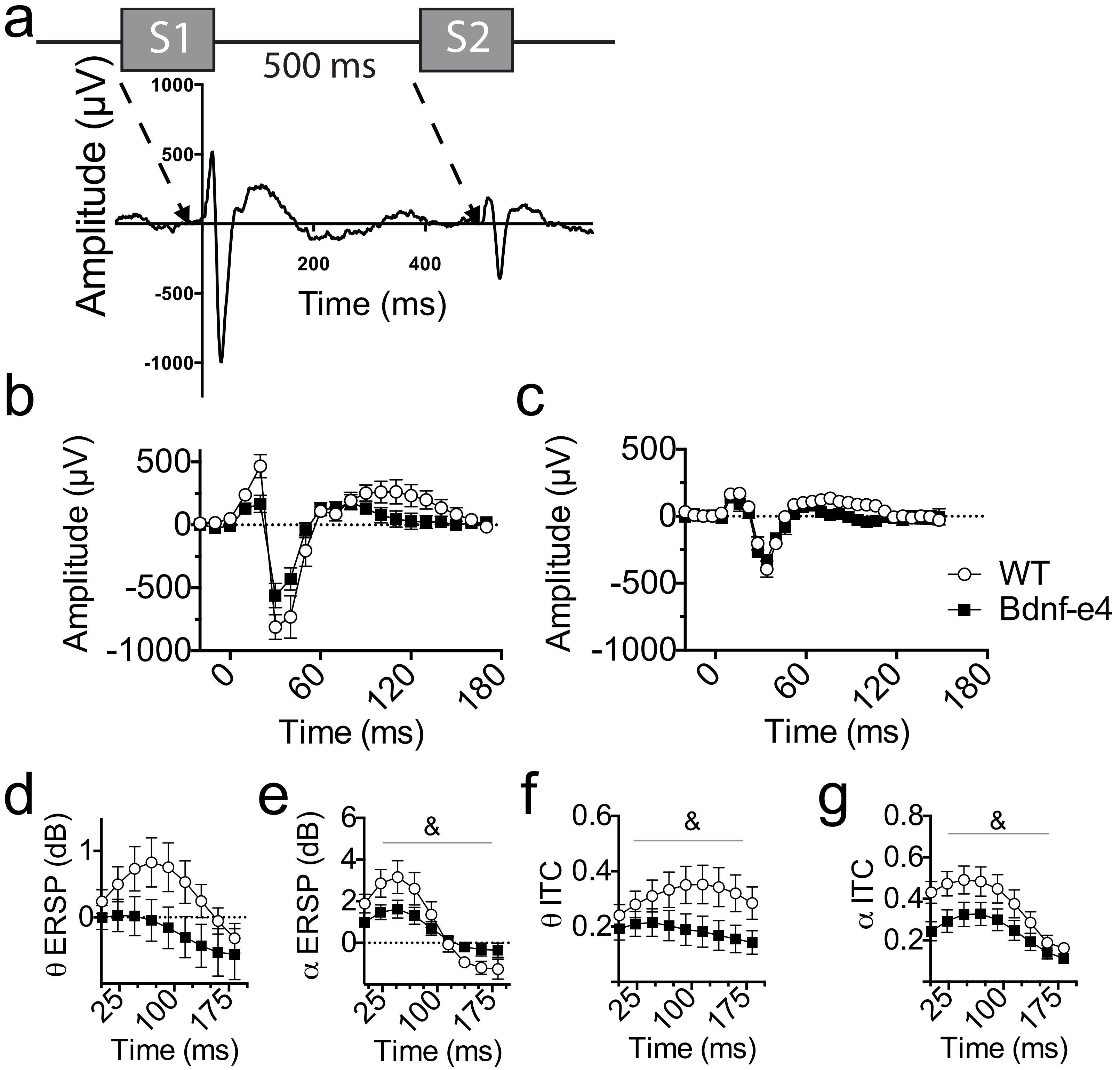
a
S1
S2
500 ms
Amplitude (µV)
Time (ms)
b
c
Amplitude (µV)
Time (ms)
WT
Bdnf-e4
d
e
f
g
θ ERSP (dB)
α ERSP (dB)
θ ITC
α ITC
&
Time (ms)

Figure S3

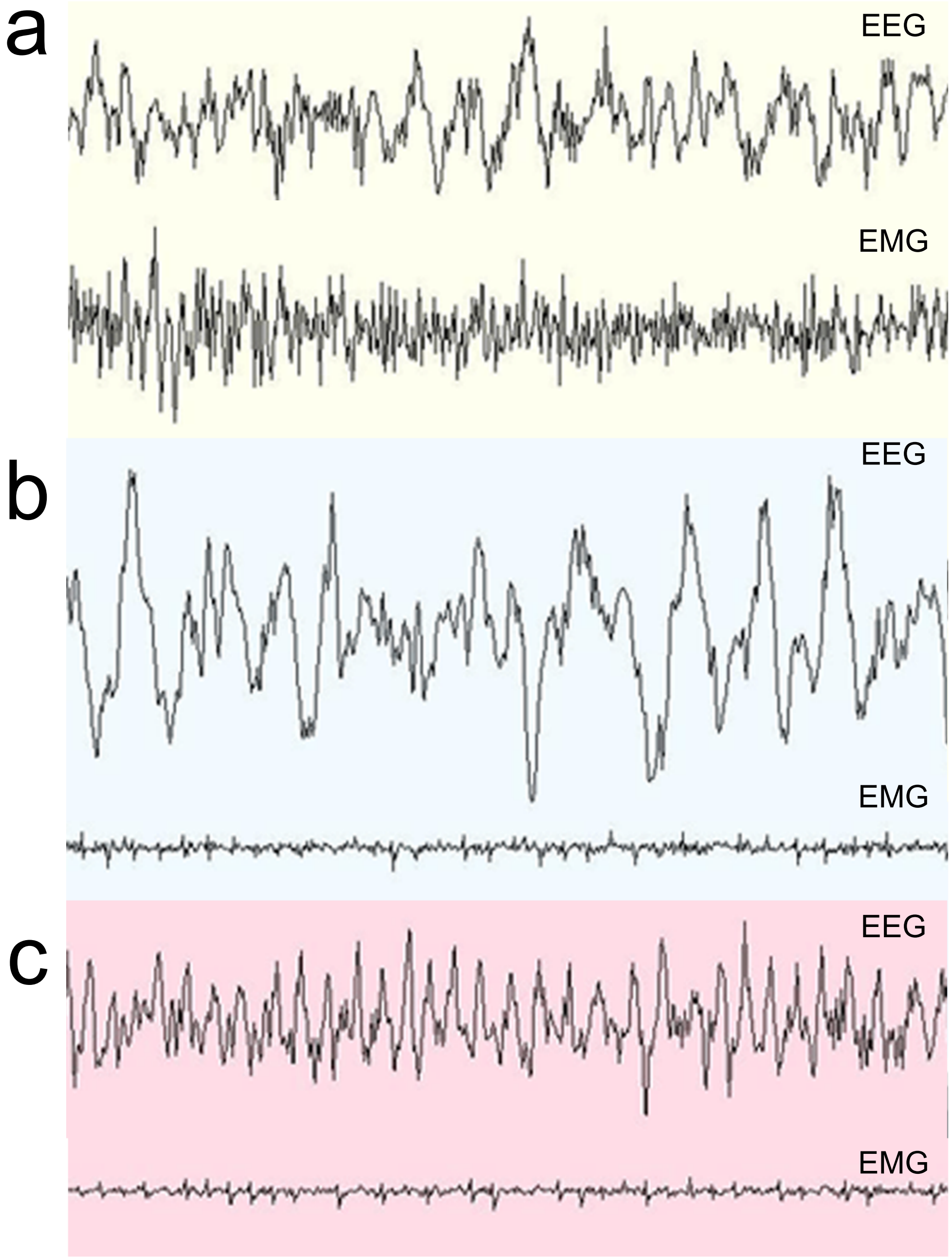

Figure S4

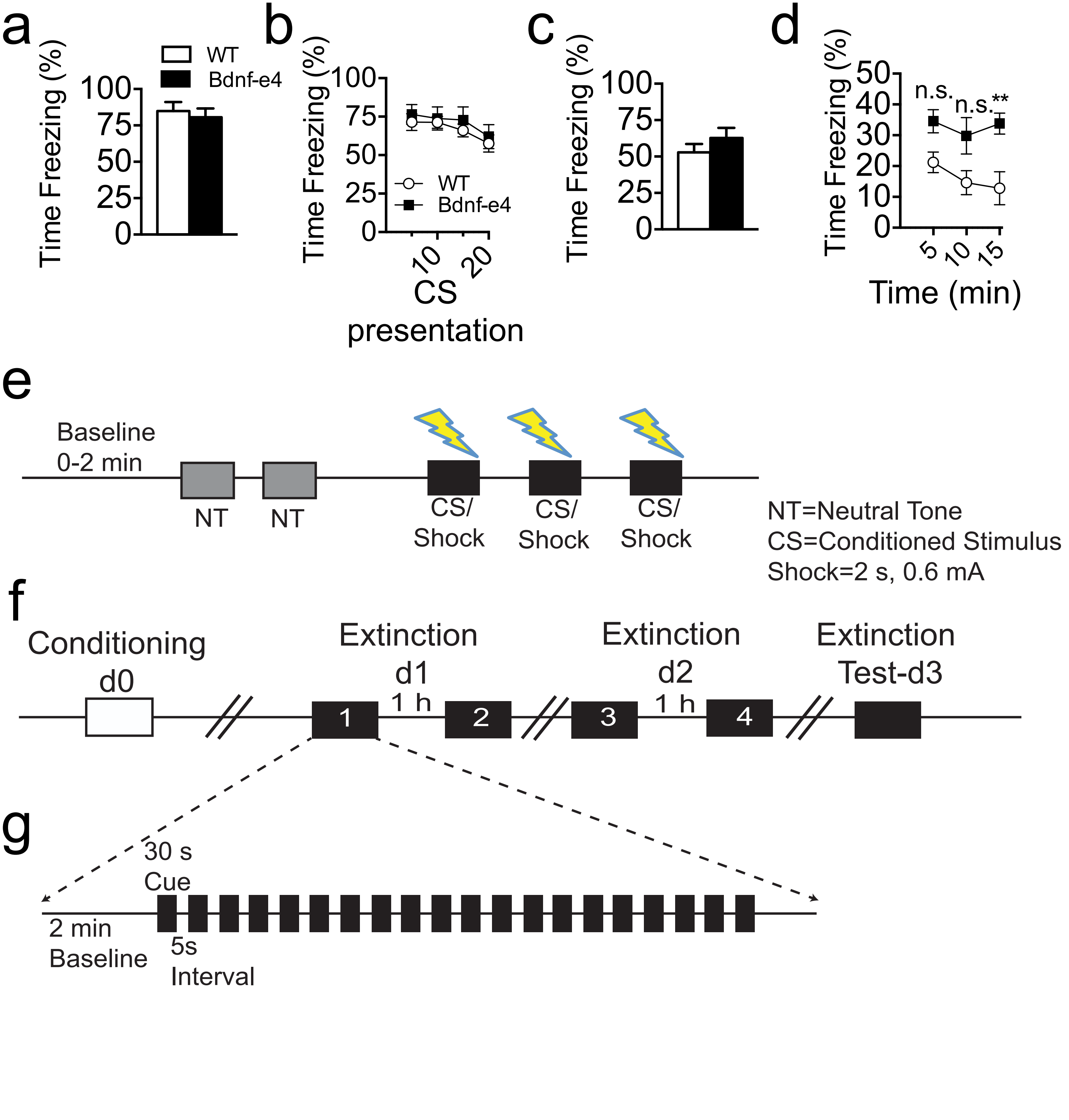
a
Time Freezing (%)
100
75
50
25
0
WT
Bdnf-e4
b
Time Freezing (%)
100
75
50
25
0
10
20
CS presentation
WT
Bdnf-e4
c
Time Freezing (%)
100
75
50
25
0
d
Time Freezing (%)
50
40
30
20
10
0
5
10
15
Time (min)
n.s.
n.s.
**
e
Baseline
0-2 min
NT
NT
CS/ Shock
CS/ Shock
CS/ Shock
NT=Neutral Tone
CS=Conditioned Stimulus
Shock=2 s, 0.6 mA
f
Conditioning d0
Extinction d1
1 h
1
2
Extinction d2
1 h
3
4
Extinction Test-d3
g
30 s
Cue
2 min
Baseline
5s
Interval

Figure S5

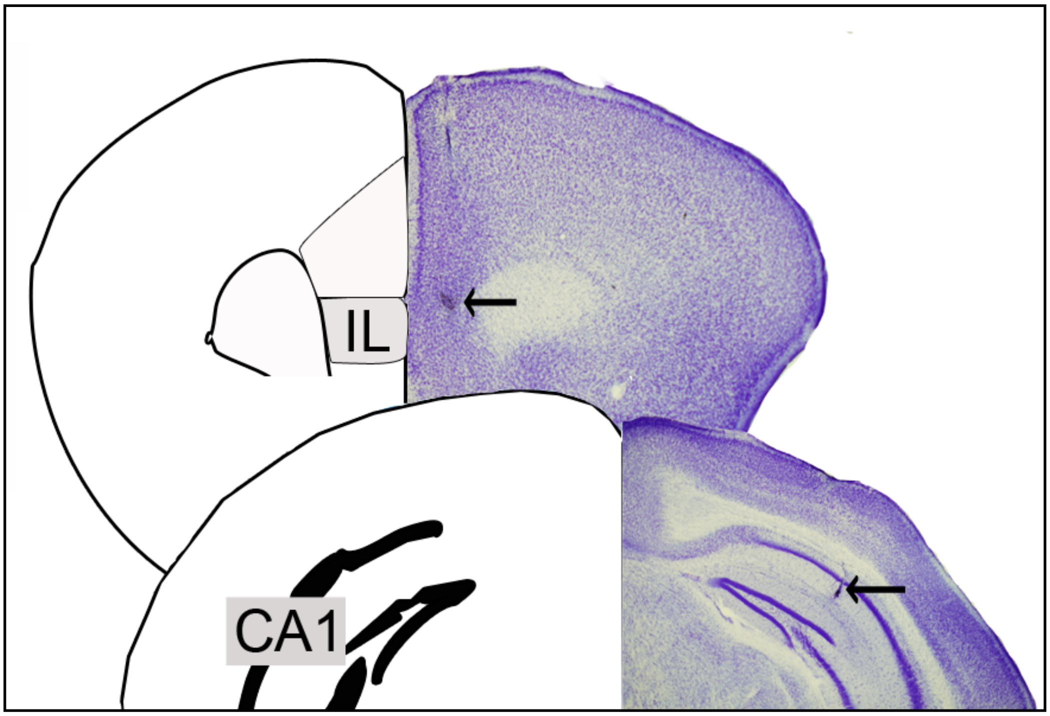

Figure S6

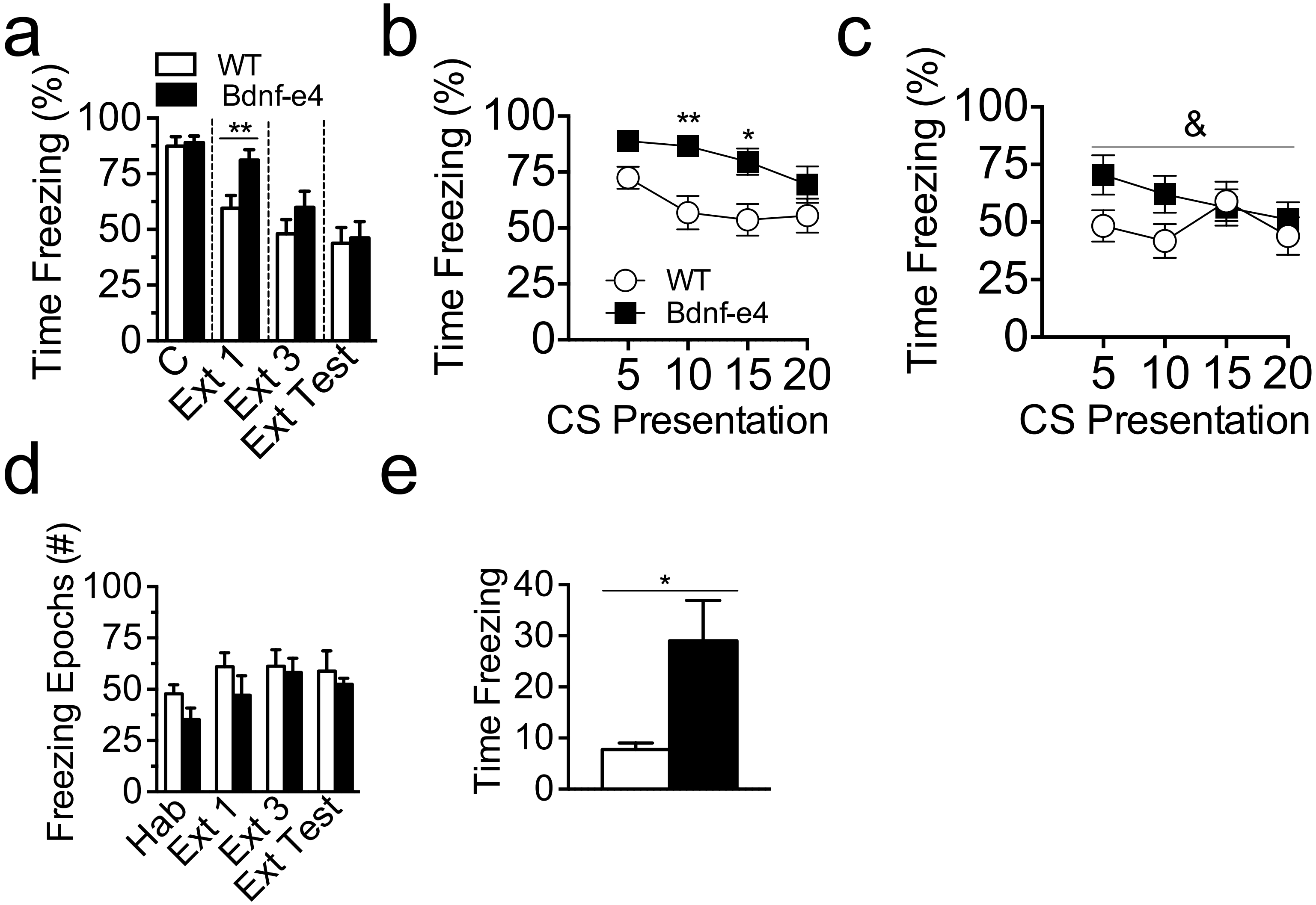
a
WT
Bdnf-e4
Time Freezing (%)
100
75
50
25
0
**
C
Ext 1
Ext 3
Ext Test
b
Time Freezing (%)
**
*
WT
Bdnf-e4
5 10 15 20
CS Presentation
c
Time Freezing (%)
&
5 10 15 20
CS Presentation
d
Freezing Epochs (#)
Hab
Ext 1
Ext 3
Ext Test
e
Time Freezing
40
30
20
10
0
*

Figure S7

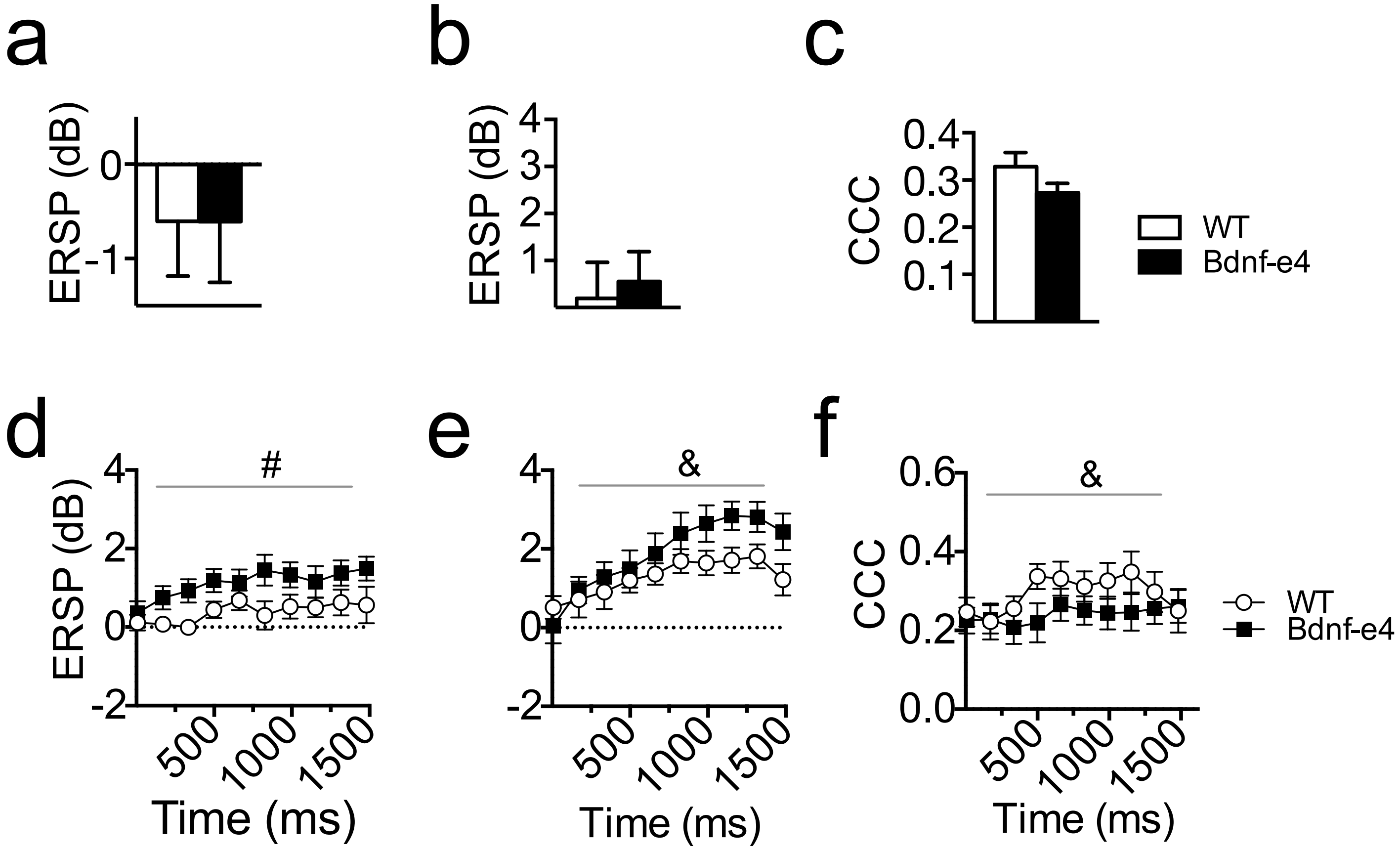

a
ERSP (dB)
0
-1
b
ERSP (dB)
4
3
2
1
c
CCC
0.4
0.3
0.2
0.1
WT
Bdnf-e4
d
ERSP (dB)
4
2
0
-2
#
500
1000
1500
Time (ms)
e
4
2
0
-2
&
500
1000
1500
Time (ms)
f
CCC
0.6
0.4
0.2
0.0
&
500
1000
1500
Time (ms)
WT
Bdnf-e4

Figure S8

Supplemental Figure S1: Bdnf-e4 mouse model. (a) Schematic of transcript production from *Bdnf* gene. Transcription initiation occurs from promoters upstream of each 5' untranslated region (UTR), and is spliced to the common coding exon (pink). Produced mRNA transcripts all produce a similar BDNF protein. (b) Schematic of the targeting vector for the original BDNF-KIV founder line (top), and the second generation Bdnf-e4 line (bottom). Note that the BDNF-KIV line retained a floxed PGK-Neo cassette, which was removed in the Bdnf-e4 mice. (c) An example of promoter IV-driven transcription and splicing in Bdnf-e4 mice. Transcription from promoter IV results in expression of a *Bdnf*-IV-eGFP-STOP-*Bdnf*-IX transcript. Translation of the produced mRNA results in GFP production in lieu of BDNF. (d) qPCR for expression of exon I, II, IV and VI-containing *Bdnf* transcripts in Bdnf-e4 mice. Results from prefrontal cortex (PFC) (left, *n*=4, each genotype) and hippocampus (HPC) (right, *n*=5, each genotype) demonstrate that exon IV-containing transcripts are absent in Bdnf-e4 mice. Downregulation of other *Bdnf* transcripts is limited to a decrease in exon I-containing transcripts in Bdnf-e4 HPC (Student's *t*-test, *p*=0.0041). (e) qPCR for interneuron marker expression in Bdnf-e4 mice. Compared to WT, Bdnf-e4 mice have a significant decrease in expression of cortistatin (*Cort*) (Student's *t*-test, *p*=0.0040), somatostatin (*Sst*) (Student's *t*-test, *p*=0.0002), and parvalbumin (*Pvalb*) (Student's *t*-test, p=0.037) in the PFC, left. In the HPC, Bdnf-e4 mice have significant decreases in *Cort* (Student's *t*-test, *p*=0.0169) *and Sst* (Student's *t*-test, p=0.0182) compared to WT*,* but there is not a significant difference in *Pvalb* expression (Student's *t*-test, p=0.3029). Data are represented as means ± SEM (*p<0.05, ***p*<0.001, ****p*<0.0001).

Supplemental Figure S2: Baseline behaviors in Bdnf-e4 mice. (a-c) No difference in anxiety-like behavior between WT and Bdnf-e4 mice (*n*=8-9/group). (a) WT and Bdnf-e4 mice spent equivalent amounts of time in the center of the open field (Student's *t*-test, *p*=0.1791) (b) the light area in the light dark box (Student's *t*-test, *p*=0.8755), and (c) the open arms of the elevated zero maze (Student's *t*-test, *p*=0.3673). (d) Locomotor function is similar in Bdnf-e4 mice compared to WT animals. Distance traveled by Bdnf-e4 mice was equivalent across time compared to WT (Two-way ANOVA, genotype x time interaction, $F_{38,532}$=1.159, *p*=0.3280), as well as (e) the total distance traveled (Student's *t*-test, *p*=0.9731). (f-g) Depressive-like behaviors are similar between Bdnf-e4 mice and WT. In the forced swim test, there was no difference in (f) the duration of time spent immobile across time (Two-way ANOVA, genotype xtime interaction, $F_{5,70}$=1.159, *p*=0.3380), or (g) the duration of immobility in the last 4 minutes of testing (Student's *t*-test, *p*=0.2732). (h) There was no difference in other routine behaviors during homecage recordings between WT and Bdnf-e4 mice, including eating, drinking, rearing, walking, or grooming.

Supplemental Figure S3: Event-related potential (ERP) experimental design and additional ERP analysis results. (a) Trial design for the ERP experiment (S1=Sound 1

and S2=Sound 2). S1 and S2 consist of identical 70 dB intensity tones separated by a 500 ms inter-stimulus interval (ISI). Dashed line represents time of stimulus onset. Below, the physiological response as measured by the amplitude of electroencephalogram (EEG) is depicted. The response to the second tone is gated, i.e. the amplitude to S2 is smaller than to S1. (b-c) Grand average responses for WT (*n*=8) and Bdnf-e4 (*n*=8) following S1 and S2, obtained by averaging the EEG response obtained for each animal. (b) Grand average response for S1. (c) Grand average response for S2. (d-e) Post-S1 event-related spectral perturbation (ERSP) results depicted as a line graph for theta (3-8 Hz) and alpha (8-12 Hz) frequencies. (d) There is not a significant difference in theta ERSP between WT and Bdnf-e4 mice (genotype x time interaction, $F_{52,728}$=1.092, p<0.3090). (e) Bdnf-e4 mice have decreased alpha ERSP response following S1 (genotype x time interaction, $F_{52,728}$=4.023, p<0.0001). (f-g) Post-S1 Inter-trial phase coherence (ITC) results for theta and alpha frequencies. (f) Theta ITC is significantly decreased in Bdnf-e4 mice (genotype x time interaction, $F_{52,728}$=4.805, p<0.0001). (g) Alpha ITC is also decreased in Bdnf-e4 mice (genotype x time interaction, $F_{52,78}$=2.384, p<0.0001). Data are means ± SEM (&=p<0.001, genotype x time interaction)

Supplemental Figure S4: Representative traces illustrate EEG/Electromyogram (EMG) activity during different behavioral states. (a-c) For each state, EEG and EMG activity is displayed in microvolts (μV). a) During wake, the EEG data consists of a range of frequency activity, and the EMG is high in amplitude. (b) Data from non-rapid eye movement (NREM) sleep. High amplitude, slow-wave activity (SWA) under 4 Hz dominates the EEG pattern, and the EMG activity has a low amplitude. (c) Data from rapid eye movement (REM) sleep. EEG waves in the REM state have increased theta frequency content and decreased amplitude compared to NREM. The EMG activity during REM is also of a low amplitude.

Supplemental Figure S5: Behavioral experiments characterizing fear extinction in Bdnf-e4 mice. (a-b) There were no differences between WT (*n*=11) and Bdnf-e4 (*n*=13) mice in fear acquisition or time spent freezing during cue-only fear extinction. (a) No difference between genotypes in time spent freezing in the last 30 s of conditioning (Student's *t*-test, p=0.6227). (b) Two-way ANOVA revealed no difference between genotypes in time spent freezing during conditioned stimulus (CS) presentation (genotype effect, $F_{1,23}$=.3821, p=0.5425). (c-d) There were no differences between WT and Bdnf-e4 mice in fear acquisition, but Bdnf-e4 mice show impaired context extinction (*n*=9, both genotypes). (c) During conditioning there was no difference in time spent freezing between genotypes in the last 30 s (Student's *t*-test, p=0.2886). (d) Bdnf-e4 mice show impaired context-only extinction compared to WT animals. Two-way ANOVA revealed a significant effect of genotype on the average time spent freezing (genotype effect, $F_{1,14}$=10.55, p=0.0058). Post-hoc analysis did not reveal significant effects until

the final 5 min of context extinction testing (1-5 min, and 5-10 min, n.s., 10-15 min 15, *p*=0.001). (e-g) Testing paradigm for the combined cue/context extinction experiments presented in Figure 3. (e) During conditioning, mice were first exposed to two 30 s, 1000 Hz neutral tones (NT) to assess any potential noise-induced freezing prior to conditioning. Conditioning consisted of 3 tone (4000 Hz)-foot-shock (0.6 mA) pairings, with the tone played for 30 s and the foot-shock taking place in the last 2 s of the tone. The conditioning session lasted 10 min. (f) 24 h following conditioning, extinction trials were started. On d 1 and d 2 mice were exposed to two sessions of extinction training (d1- Extinction 1, Extinction 2, and d 2- Extinction 3, Extinction 4, noted by white numbers) separated by a 1 h interval. 72 h post-conditioning on d 3 mice performed an Extinction Test. Hatched lines between sessions indicate a 24 h break between sessions. (g) Each extinction session consisted of a 2 min baseline in the conditioning context, followed by 20 CS tone exposures, which were 30 s in length with a 5 s ISI. Extinction sessions were 14.5 min long. Data are means ± SEM (***p*<0.001, n.s.=not significant).

Supplemental Figure S6: Representative histology from animals with depth electrodes targeted to the infralimbic (IL) region of medial PFC (mPFC) and CA1 region of HPC. Following perfusion, brains were sectioned and cresyl violet stained. Representative examples of IL and CA1, with arrows pointing to the site of the electrode track.

Supplemental Figure S7: Behavioral results acquired from local field potential (LFP) recordings conducted during extinction (a-c) Behavior results during electrophysiological recordings (WT *n*=11; Bdnf-e4 *n*=13). (a) Average time spent freezing per session, during Conditioning (C), Extinction 1 (Ext 1), Extinction 3 (Ext 3), and Extinction Test (Ext Test). During Extinction 1, the session in which HPC theta power is higher and cross channel coherence (CCC) is reduced in Bdnf-e4 mice, Bdnf-e4 mice freeze significantly more than WT (Student's *t*-test, *p*=0.0075). (b) For Extinction 1, time spent freezing is displayed, with each point representing the average freezing during 5 CS presentations. There was a significant effect of genotype on freezing during Extinction 1, as shown by a two-way ANOVA (genotype effect, $F_{1,22}$=8.612, *p*=0.0077). Post hoc analyses revealed significant differences during the middle of the extinction session (CS 1-5 and 16-20, n.s.; CS 6-10, *p*=0.001, CS 11-15, *p*<0.05). (c) For Extinction 3, time spent freezing averaged across 5 CS presentations is displayed. There is a significant genotype x time interaction during Extinction 3 for Bdnf-e4 compared to WT (genotype x CS presentation interaction, $F_{3,66}$= 3.384, *p*=0.0232). (d) The number of epochs used for ERSP, ITC, and CCC analysis was not significantly different between genotypes within each session. (e) Averaged time spent freezing in Extinction 1 by number of freezing events for each animal demonstrates that Bdnf-e4 mice freeze for significantly longer during each freezing event (Student's *t*-test,

p=0.0184). Data are represented as means ± SEM (**p*<0.05, ***p*<0.001, &=genotype-time interaction, *p*<0.05).

Supplemental Figure S8: Additional physiological data from recordings conducted during extinction. (a-c) ERSP and CCC representing electrophysiological results during the Habituation session. Data is averaged from 3-5 Hz (low theta), and is taken from 0-1000 ms following cessation of motion. (a) ERSP data from the mPFC (*n*=10 each genotype) and (b) ERSP data from the HPC (*n*=10 WT; *n*=12 Bdnf-e4) demonstrate that there are no differences between genotypes during Habituation in the low theta frequency range. (c) Additionally, there is not a significant difference between genotypes during habituation in HPC-mPFC phase synchrony, measured by CCC (*n*=9 WT; *n*=10 Bdnf-e4). (d-f) Line graph representation of data averaged from 3-5 Hz during Extinction 3 from 0-1630 ms post-freezing demonstrates subtle differences in WT and Bdnf-e4 ERSP and CCC data across time. (d) For the mPFC (*n*=10 WT; *n*=11 Bdnf-e4), there is a significant genotype difference in low theta power during Extinction 3 as revealed by a two-way ANOVA (genotype effect, $F_{1,20}$=6.406, *p*=0.0199). (e) For the HPC (*n*=10 WT; *n*=13 Bdnf-e4), (genotype x time interaction, $F_{99, 2079}$=1.263, *p*=0.0437). (f) For low theta phase synchrony (*n*=10 WT; *n*=11 Bdnf-e4), there is a significant genotype x time interaction during Extinction 3, as evidenced by a two-way ANOVA (genotype x time interaction, $F_{99,1782}$=1.669, *p*<0.0001). There were no specific time points that reached significance in the post-hoc testing. Data are means ± SEM (#=genotype difference, *p*<0.05; &=genotype x time interaction, *p*<0.05).

Supplementary Methods

<u>Quantitative PCR (qPCR)</u>

qPCR was conducted as previously described[1]. Briefly, WT and Bdnf-e4 adult mice (n=4-5 per genotype) were cervically dislocated and brain tissues were snap frozen with isopentane- this tissue was used for both qPCR and ELISA. Total RNA was isolated from hippocampus (HPC) and PFC (PFC) using TRIzol (Life Technologies, Carlsbad, CA). RNA was purified using an RNeasy minicolumn (Qiagen, Valencia, CA) and quantified by a NanoDrop spectrophotometer (Agilent Technologies, Savage, MD). Using Superscript III (Life Technologies), RNA concentration was normalized and reverse transcribed into single-stranded cDNA (Life Technologies). Quantitative PCR was performed using a Realplex thermocycler (Eppendorf, Hamburg, Germany) using

GEMM mastermix (Life Technologies) with 40 ng of synthesized cDNA. Individual mRNA levels were normalized for each well to *Gapdh* mRNA levels.

Homecage Behavior Monitoring

Homecage recordings were conducted as described previously[2]. Mice were placed in a home cage environment in a sound-attenuated, temperature- controlled chamber. Recordings using digital cameras were begun at 6 pm, at the beginning of the dark cycle. Infrared light was used for illumination during the dark cycle. Automated video analysis of homecage behavior was completed using HomeCageScan (HCS) software (Cleversys Inc., Reston, VA). Using HCS, detection of behavior occurs by utilizing information about whole-body movements of the mouse- animal body parts such as head, tail, and limbs are identified, and then sequences of data are used to automatically analyze animal behavior in durations > 6 frames.

Testing of Baseline Behaviors

Prior to all behavioral testing, mice were habituated for at least one hour in a quiet room with low lighting. Open field, Light dark box testing, and elevated zero results were all assessed using Topscan behavioral software from CleverSys, Inc. Video was recorded from above, with a “bird’s eye” view. *Open field testing-* Mice were placed in the center of an 18”x18” box. Distance travelled and duration spent in the corner and center within the arena of the box was tracked. *Light dark box-* Mice were placed into a 10”by17” box with an enclosed, dark space area and a light, open area to assess anxiety. Frequency and duration of time spent in both the light and dark areas of the box were recorded. *Elevated zero maze-* Mice were placed, one at a time, in the open arc of the elevated zero maze. The frequency of the animal’s crossing between the open and closed arms, as well as the length of time spent in the open and closed arms, was calculated. *Forced*

swim test-mice were placed in a warm water tank for 6 minutes, and the bouts and duration of immobility were measured. The last 4 minutes was used for the total duration measurement, to examine escape-related behavior.

Cued and Contextual Extinction

For all experiments, WT and Bdnf-e4 -/- mice were conditioned as described in the main text, and in Fig S4e. During conditioning, mice were first exposed to two 30 s, 1000 Hz neutral tones (NT) to assess any potential noise-induced freezing prior to conditioning. Conditioning consisted of 3 tone (4000 Hz)-foot-shock (0.6 mA) pairings, with the tone played for 30 s and the foot-shock taking place in the last 2 s of the tone. For cue and context extinction, mice were extinguished 24 h post-conditioning. For cue extinction, the interior of the conditioning chamber was altered with novel spatial cues and the animals were set in a plastic container, which covered the metal floor bars associated with foot shock. An identical auditory protocol was used for cue extinction that was used for the cue/context paradigm. For context extinction, mice were placed back in the conditioning chamber for 15 min and time spent freezing for each min was scored, and then averaged across 5 min.

1 Maynard KR, Hill JL, Calcaterra NE, Palko ME, Kardian A, Paredes D *et al.* Functional Role of BDNF Production from Unique Promoters in Aggression and Serotonin Signaling. *Neuropsychopharmacology* 2016; **41**: 1943–55.

2 Martinowich K, Schloesser RJ, Jimenez D V, Weinberger DR, Lu B. Activity-dependent brain-derived neurotrophic factor expression regulates cortistatin-interneurons and sleep behavior. *Mol Brain* 2011; **4**: 11.
